# ASCEND: A Study of Cardiovascular Events iN Diabetes: Characteristics of a randomized trial of aspirin and of omega-3 fatty acid supplementation in 15,480 people with diabetes

**DOI:** 10.1016/j.ahj.2017.12.006

**Published:** 2018-04

**Authors:** Louise Bowman, Marion Mafham, William Stevens, Richard Haynes, Theingi Aung, Fang Chen, Georgina Buck, Rory Collins, Jane Armitage, The ASCEND Study Collaborative Group

**Affiliations:** a Clinical Trial Service Unit, Richard Doll Building, Old Road Campus, Roosevelt Drive, Headington, Oxford; b Medical Research Council Population Health Research Unit, Nuffield Department of Population Health, University of Oxford, Oxford, England; c Department of Endocrinology, Royal Berkshire Hospital, Reading, UK

## Abstract

**Objectives:**

The use of aspirin for the secondary prevention of cardiovascular disease (CVD) is firmly established, and the proportional reductions in heart attacks and strokes appear to be similar in people with and without diabetes. Uncertainty remains about the role of antiplatelet treatments for primary prevention of CVD, and guidelines vary in their recommendations. It has also been hypothesized that long-term aspirin can prevent gastro-intestinal and other cancers.

Observational studies suggest associations between higher intakes of omega-3 fatty acids (FA) and lower rates of CVD, but there is no large-scale randomized evidence to support using prophylactic omega-3 FA supplementation in primary prevention.

ASCEND is a randomized trial assessing whether 100 mg daily aspirin safely prevents CVD and cancer in patients with diabetes without known arterial disease. It is also assessing whether supplementation with 1 g omega-3 FA daily prevents CVD. This paper describes the methods and baseline characteristics of the randomized participants.

**Methods and results:**

Between 2005 and 2011, using mail-based methods, 15,480 people with diabetes were randomized to aspirin versus placebo and, in a factorial design, to omega-3 FA supplementation versus placebo. Blood and urine samples were collected to allow baseline stratification by biochemical prognostic variables (e.g. HbA1c, blood lipids). Follow-up is for a median of at least 7 years.

**Conclusions:**

Demonstrating that prophylactic aspirin safely reduces the risk of CVD or cancer in the primary prevention setting, or that omega-3 FA supplementation prevents CVD, would be relevant to hundreds of millions of people worldwide who are currently not receiving such therapies. The results of ASCEND will be reported in 2018.

ASCEND is a randomized placebo-controlled trial aiming to determine whether 100 mg daily aspirin prevents cardiovascular events or cancer in 15,480 UK patients with diabetes who are not already known to have occlusive arterial disease, without leading to significant bleeding or other adverse events that outweigh any benefits. It is also assessing whether supplementation with 1 g omega-3 fatty acids (FA) daily prevents CVD.

## Aspirin in primary prevention

The Anti-Thrombotic Trialists' Collaboration (ATTC) demonstrated conclusively that antiplatelet therapy (chiefly aspirin) reduces the risk of myocardial infarction (MI), stroke or cardiovascular death by about one-quarter in people with occlusive vascular disease, including among those who have diabetes.^1^ However, most of the 3 million people with diabetes in the UK, and the estimated 400 million worldwide,^2^ do not have manifest vascular disease. The 2009 ATTC individual-patient-data meta-analysis of 95,000 patients in 6 primary prevention trials found that allocation to aspirin yielded a 12% (95% CI, 6%-18%) proportional reduction in occlusive vascular events, mainly due to a reduction in non-fatal MI of about one fifth.^3^ However, given the approximate 50% proportional increase in the risk of bleeding with aspirin, on average the bleeding hazard counterbalanced much of the benefit in these low-risk primary prevention patients. Among the participants in these primary prevention trials, only about 4% had diabetes and the relative risk reduction among them was similar to that observed in those without diabetes. Consequently, since people with diabetes are generally at 2- to 3-fold higher risk of vascular events than those without it,^4^ the absolute risk reduction with aspirin is likely to be greater than for healthy volunteers. However, the ATTC analyses also found that people with diabetes had a higher risk of both major extra-cranial bleeds (rate ratio [RR], 1.55; 95% CI 1.13–2.14) and of hemorrhagic stroke (RR, 1.74; 95% CI, 0.95-3.17, respectively) compared to those who did not have diabetes irrespective of aspirin allocation.^3^

A further four trials of aspirin for primary prevention of cardiovascular events have reported results since the ATTC analyses were published in 2009: two specifically in diabetes,5., 6. and two in wider populations that included people with diabetes.7., 8. The Prevention of Progression of Arterial Disease and Diabetes (POPADAD) trial in 1276 patients with diabetes and reduced ankle-brachial index observed no effect on vascular events over 6.7 years (18.2% vs 18.3%; HR, 0.98; 95% CI, 0.76-1.26), while the Japanese Primary Prevention of Atherosclerosis With Aspirin for Diabetes Trial (JPAD) in 2539 patients with type 2 diabetes and no history of atherosclerotic disease followed for 4.4 years observed a non-significant reduction in vascular events based on a very low event rate (1.36% vs 1.70%; HR, 0.80; 95% CI, 0.58-1.10). Neither of these trials reported detailed information about bleeding, so the balance of benefits and risks with aspirin use for primary prevention in diabetes remains uncertain.

### Aspirin in primary prevention of cancer

Recent retrospective meta-analyses of randomized trials have suggested that aspirin may produce 15% to 20% proportional reductions in cancer incidence or death, with 30% to 40% reductions in gastrointestinal cancers (particularly colorectal cancer), and that these effects increase with more prolonged exposure.9., 10., 11., 12., 13. If such effects on cancer are confirmed prospectively in randomized trials of sufficient duration, they could have significant implications for the balance of benefits and hazards of using aspirin for primary prevention. ASCEND provides the opportunity to test this hypothesis prospectively with good statistical power since there about as many incident cancers (approximately 1500 during the scheduled treatment period) as in the meta-analyses that generated the hypothesis of protection against cancer and it involves prolonged exposure to aspirin (a median of at least 7 years), with longer-term follow-up available from central registers.

### Omega-3 fatty acids in diabetes: adding to the randomized evidence

A possible link between intake of omega-3 fatty acids (FA) and prevention of coronary heart disease (CHD) was first noted in the 1940s when the diets of Greenland Eskimos, among whom CHD was rare despite a high fat intake largely due to sea food, were compared with those of Danes in Denmark who had similarly high fat intake from more mixed diets but CHD rates that were about 10 times higher.^14^ A large number of observational studies of omega-3 FA intake and heart disease risk were subsequently conducted in different populations. A systematic review of these observational data concluded that consumption of the equivalent of 40–60 grams of fish per day (providing about 0.2–1 g daily of omega-3 FA, depending on the type of fish) is associated with about a halving in rates of cardiac death.^15^ However, there were only limited data available from randomized controlled trials of the effects of increasing omega-3 FA intake on cardiovascular disease outcomes. The results in one small randomized trial involving 2000 male heart attack survivors were consistent with the observational studies, with a 29% (95% CI, 7–46%) significant reduction in total mortality and a 16% (95% CI, +7 to 24%) non-significant reduction in ischemic heart disease events.^16^ Similarly, in a trial of omega-3 FA (1 g daily) among 11,000 patients who had survived a myocardial infarction, there was a 13% (95% CI, 1–24%) proportional reduction in coronary events but, both this and the 10% (95% CI, 1–18%) reduction in the primary outcome of cardiovascular events, were only marginally significant.^17^ As a consequence, several large randomized trials (including the present ASCEND trial) were started in order to generate more reliable evidence about the effects of omega-3 FA supplementation. Some of those trials have now reported their results, and combined in a tabular data meta-analysis of 10 such trials that each included at least 500 participants treated for at least 1 year involved over 11,000 vascular events in 78,000 participants.^18^ Allocation to omega-3 FA supplements (weighted mean daily dose of 1.1 g) for an average duration of 4.4 years appeared to have no significant effect on major vascular events, either overall (RR 0.97; 95% CI 0.93-1.01) or in any particular subgroup. ASCEND will contribute important additional data on both efficacy and safety of such supplementation and, given its large size and longer duration than any previous trial, may be able to detect any modest effects of omega-3 FA supplementation.

## Methods

### Objectives

The aim of ASCEND is to determine whether daily 100 mg aspirin prevents cardiovascular events or cancer in patients with diabetes who are not known to have occlusive arterial disease, as well as to assess the magnitude of any effects on significant bleeding or other serious adverse events. It is also assessing whether supplementation with daily 1 g capsules containing 90% omega-3 fatty acids (0.41 g eicosapentaenoic acid, 0.34 g docosahexaenoic acid) prevents CVD.

### Eligibility

Men or women aged at least 40 years at the time of invitation for screening were eligible for the study, provided they fulfilled all of the following criteria:

i) *Clinical diagnosis of diabetes mellitus:* the participant's own doctor considered them to have type 1 or type 2 diabetes (based on standard WHO or ADA diagnostic criteria19., 20.);
ii) *No clear indication for aspirin:* the participant had no diagnosed occlusive arterial disease (i.e. a history of MI, angina pectoris, coronary or non-coronary revascularization procedure [ie, peripheral arterial bypass surgery or angioplasty], stroke or transient ischemic attack);
iii) *No clear contra-indication to aspirin:* The participant was not at high risk of bleeding due to gastrointestinal hemorrhage or peptic ulcer within the previous 6 months, active hepatic disease (such as cirrhosis or active hepatitis), or use of warfarin or other anti-coagulant therapy; and had no history of aspirin allergy;
iv) *Substantial uncertainty about whether antiplatelet or omega-3 FA therapy confers worthwhile benefit:* the participant and their own general practitioner (GP) did not consider there to be a definite need to use aspirin or omega-3 FA supplements regularly (or a definite need not to do so);
v) *No other predominant life-threatening medical problem:* the participant did not have some condition (other than diabetes) that might be expected to prevent them from taking at least 5 years of study treatment.

### Participant recruitment and follow-up

In order to be cost-effective, UK-wide recruitment into ASCEND was conducted by mail. The highly streamlined recruitment methods have been described previously.^21^ The coordinating center provided a 24-hour Freefone service to answer questions about the trial from participants and their GPs.

#### Invitation and screening

In collaboration with medical consultants and GPs around the UK, and supported by the National Institutes for Health Research (NIHR) Diabetes and Primary Care Research Networks (DRN and PCRN), potentially eligible patients with diabetes were identified from centrally-held registers (e.g. for retinopathy screening) and GP-held disease registers. Potential study participants were mailed an invitation pack, including a cover letter, screening questionnaire (to determine eligibility and to seek consent), Freepost envelope and Information Leaflet.

#### Pre-randomization run-in period

Willing and eligible patients entered a pre-randomization run-in phase and were sent a run-in pack of medication (single blind: containing placebo aspirin tablets and placebo omega-3 FA capsules) and asked to take one tablet and one capsule daily for 2 months. During the run-in period, the participant's GP was informed by letter of their patient's possible involvement in the study and asked to return a form if they considered there to be any reason not to randomize their patient. Patients were randomized only if, at the end of the run-in period, they seemed likely to comply with the study protocol for several more years. By this process, many potential dropouts could be excluded before becoming part of the randomized comparison, with a consequent improvement in statistical sensitivity of the “intention-to-treat” analyses.^22^

#### Randomization

About 2 months after starting the run-in, participants were sent a more detailed randomization questionnaire asking about any significant problems (including any cardiovascular events), their compliance with the study treatments during the run-in period, details of their diabetes history (to allow classification as type 1 or 2),^23^ current medication, ethnic group and smoking history.

Participants who remained eligible based on the randomization questionnaire and were willing to continue on the study were randomized centrally at the Clinical Trial Service Unit (CTSU), University of Oxford, using a minimization algorithm to ensure balance by prognostic variables (age, sex, duration of diabetes, history of treated hypertension, smoking status, ethnic origin, and, if available from centrally measured blood and urine samples [see below], total cholesterol, HbA1c, and urinary albumin/creatinine ratio). Eligible patients were randomized in a 2 × 2 factorial blinded design between aspirin 100 mg daily and matching placebo, and, separately, between omega-3 fatty acid capsules 1 g daily and placebo.

#### Post-randomization follow-up

Follow-up was also conducted largely by mail, supplemented by information from central registries. Randomized participants received a follow-up questionnaire 6-monthly (either paper or via a weblink to a secure online version^24^) asking about the occurrence of any cardiovascular events, bleeding events, cancer diagnoses, compliance with study medication and use of other relevant medications (such as anti-platelet agents or anti-coagulants). Confirmation and further information was then sought from GPs about reports of possible cardiovascular events and serious bleeds. All such information is reviewed by coordinating center clinicians, blind to treatment allocation, and events are adjudicated according to pre-specified criteria. Additional follow-up for death, cancer and hospitalizations is being obtained from NHS Digital (formerly the Health and Social Care Information Centre) in England and Wales and the Information Services Division of NHS Scotland. Ethics approval has been obtained for additional follow-up after the scheduled treatment period via these central registries to assess the longer-term effects on cancer and on other outcomes.

### Central biological sample assays

About 2 to 4 weeks after entering the pre-randomization run-in period, participants were sent an optional blood and urine sampling kit, and asked to take it to their general practice or other usual phlebotomy service for sample collection. The kit was sent with an information leaflet explaining the reasons for sample collection, a consent form for sample storage and assay (which included a section for recording blood pressure, pulse, height and weight measured by the practice nurse), a letter for the practice nurse with instructions for sample collection, and barcoded labels for the sample tubes. The completed consent form and blood (EDTA whole blood) and urine samples were to be mailed to the central laboratory at CTSU. Previous transport studies have demonstrated that a wide range of analytes (including HbA1c, lipids and cystatin C as a measure of renal function) and genetic polymorphisms can be reliably measured in whole blood samples despite delayed separation.25., 26.

Blood levels of total cholesterol, HDL-cholesterol, apolipoprotein-B, apolipoprotein-A1, HbA1c and cystatin C, and urinary creatinine and albumin were assayed (see Supplementary Appendix 1 for methods) in the central CTSU laboratory, which is a UKAS accredited testing laboratory. Aliquots of plasma, urine, red cells and DNA-containing buffy coat from all participants who provided samples have been stored in liquid nitrogen for future analyses (consistent with consent provided by participants).

### Sample size and predicted number of events

When ASCEND was designed in 2003, it was anticipated that 10,000 participants followed for 5 years with an expected 2% annual rate (based on previous trials in similar populations) of the composite primary efficacy outcome of non-fatal MI, non-fatal stroke or vascular death (excluding confirmed intracranial hemorrhage) would provide sufficient power to detect a relative reduction in vascular risk of 20–25%. However, as specified in the protocol, the Steering Committee monitored the overall (i.e. blinded) vascular event rate among the randomized participants in order to decide whether the original sample size assumptions remained valid or whether changes might be needed.

Based on information that subsequently became available from the ATTC meta-analysis of primary prevention trials,^3^ a relative reduction in the risk of occlusive vascular events with aspirin therapy seemed more likely to be only about 12% to 15%. In addition, the rate of the composite primary outcome observed among the first few thousand randomized participants during the first few years of the study was significantly lower than anticipated, at around 0.6% per annum. Consequently, the Trial Steering Committee decided to modify the trial design blind to any treatment related results in the following ways:

#### Inclusion of transient ischemic attacks (TIA) in the primary efficacy outcome

Patients are now routinely started on aspirin after a TIA,^27^ so its inclusion in the primary efficacy outcome increases the chances of detecting any effects of aspirin on cerebrovascular events (rather than having them diluted by post-TIA treatment).

#### Increase in sample size

The availability of large regional retinopathy registers from which potential participants could be invited provided an opportunity to increase the study population to 15,000 in a cost-effective manner.

#### Increase in study duration

It was possible to secure additional funding and drug supplies to extend median duration from 5 to at least 7 years.

Revised power calculations based on a 1.3% per annum rate of the revised primary efficacy outcome of serious vascular events (SVE: defined as non-fatal MI, non-fatal stroke, or vascular death, excluding confirmed intracranial hemorrhage but including TIA) indicate that ASCEND has good statistical power (ie, >90% at 2*P* < .05) to detect a 15% proportional reduction during extended follow-up of 7.5 years. The expected 1300 incident vascular events will approximately double the information currently available about the effects of using aspirin for primary prevention in people with diabetes. Consequently, inclusion of these data in an updated meta-analysis should help determine whether there are particular types of diabetic patient (eg, those at higher vascular risk) who would benefit.

### Planned comparisons of outcome

For aspirin therapy, the primary efficacy comparison will involve log-rank analyses of SVEs during the scheduled treatment period among all those allocated aspirin tablets versus all those allocated placebo tablets (ie, “intention-to-treat” comparisons). Similarly, for the omega-3 fatty acid supplementation, the primary efficacy comparison will involve log-rank analyses of SVEs during the scheduled treatment period among all those allocated omega-3 fatty acid capsules versus all those allocated placebo capsules. No allowance will be made for multiple hypothesis testing in these 2 separate primary comparisons (see Data Analysis Plan).

A key secondary outcome for aspirin will be the incidence of gastrointestinal tract cancers during the scheduled treatment period. However, little or no treatment effect is expected before about 3 years,^9^ limiting the statistical power to detect plausible effects of aspirin during the scheduled treatment period. There are expected to be ~430 GI tract cancers during the 7.5 years of follow-up. These numbers provide ~86% power at 2*P* < .05 to detect a 40% reduction in risk and 60% power at 2*P* < .05 for a 30% reduction in risk. Analyses excluding the first 3 years of follow-up are prespecified to assess whether effects are increasing with time from randomization. However, the main focus of the cancer analyses will be during longer term follow-up, when there will be much better power to detect plausible differences between the arms due to larger numbers of events. At about 5 years after the scheduled treatment period, there will be >90% at 2*P* < .01 to detect a 30% or greater risk reduction and >90% at 2*P* < .05 to detect a 25% reduction in any GI tract cancer risk.

The key safety outcome for aspirin will be any major bleed, defined as any confirmed intracranial hemorrhage (including intracerebral, subarachnoid, subdural or any other intracranial hemorrhage), sight-threatening eye bleeding, or any other serious bleeding episode (i.e. requiring hospitalization or transfusion, or fatal or disabling). Further details regarding pre-specified comparisons and statistical methods are provided in the Data Analysis Plan (see Supplementary Appendix 3).

### Organization and funding

The University of Oxford is the academic sponsor of ASCEND. The study is funded by a grant to the University of Oxford (a Special Project Grant from 2003 to 2008 (SP/03/002), followed by two renewals of the grant in 2009-2013 (SP/08/010/259) and 2015-2019 (SP/14/3/31114) from the British Heart Foundation (BHF) to cover the administrative and coordination costs of the trial. A separate BHF project grant (PG/05/013/18296) was obtained for the addition of blood and urine sampling to the study protocol. Aspirin and matching placebo are being provided by Bayer AG, and omega-3 fatty acid and matching placebo capsules by Mylan EPD (formerly by Abbott Product Operations AG and Solvay Pharmaceuticals GmbH), with funding from each company to cover drug packaging. The MRC Population Health Research Unit within the Clinical Trial Service Unit (CTSU) at Oxford University supports some study staff and receives additional funding from the British Heart Foundation and Cancer Research UK. Staff from the National Institute for Health Research Clinical Research Network, and the Scottish Primary Care Research Network, assisted with recruitment activities. The study was designed, and has been conducted, analyzed, interpreted and reported, independently by CTSU. The study is overseen by an independent Steering Committee, including UK diabetologists, clinical trialists, statisticians and representatives from the BHF. Representatives from Bayer AG and Mylan attend Steering Committee meetings as non-voting observers. The authors are solely responsible for the design and conduct of this study, all study analyses, the drafting and editing of the paper and its final contents. The first and last authors act as guarantors for this work.

## Results

### Ethics, regulatory, and research governance approvals

Multi-centre Research Ethics Committee (MREC) approval was granted for ASCEND by the North West MREC. Doctors and Dentists Exemptions (DDXs) for the use of aspirin and omega-3 fatty acids in ASCEND were obtained from the Medicines and Healthcare products Regulatory Agency (MHRA) prior to 1 May 2004. These DDXs were automatically converted to clinical trial authorizations following the implementation of the Medicines for Human Use (Clinical Trials) Regulations 2004. A separate application for research governance approval was made to all relevant NHS Trusts (both Hospital Trusts and Primary Care Trusts (PCTs), including Local Health Boards (LHBs) in Wales and Scotland).

### Participant recruitment

A total of 423,403 potentially eligible individuals were invited via the different routes of recruitment,^21^ of which 29% (121,254 people) returned a screening questionnaire. About two-thirds of those who responded declined to join the trial and a further 14,000 did not meet the eligibility criteria (Table I). After review of the questionnaire data, 26,462 participants (6% of those originally invited) were willing and eligible to join ASCEND and entered the 2-month run-in period (Figure).

**Figure:**
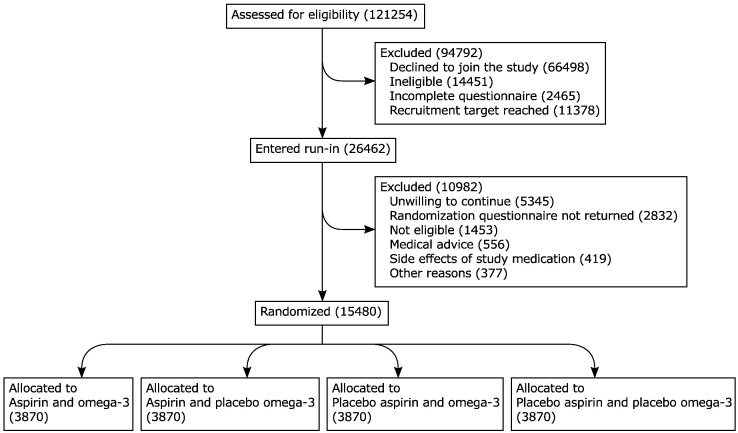
Trial profile: Flow of participants through the ASCEND trial.

**Table I:** Reasons for not entering run-in at screening.

**Reason for not entering run-in**	**Number of patients**
Declined to join the study	66,498 (70%)
Ineligible at screening*	14,004 (15%)
Prior coronary artery disease	6406
Declined to stop pre-study aspirin	3928
Declined to avoid non-study aspirin	3657
Prior stroke or transient ischemic attack	2374
On warfarin/acenocoumarol/phenindione	1312
Allergic to aspirin or omega-3	1224
Cancer in the last 5 years	871
Gastrointestinal bleeding in the last 6 months	780
Active peptic ulcer in the last 6 months	605
Did not have diabetes	501
Other serious illness	408
Prior non-coronary revascularization	230
Liver disease	158
Too young†	22
Incomplete questionnaire - unable to process	2465 (3%)
Potentially eligible at screening but subsequently ineligible‡	447 (<1%)
Screening form not processed as recruitment target reached	11,378 (12%)
Total (completed a screening form but did not enter run-in)	94,792 (100%)

* More than one reason may apply per patient.

† This includes some individuals who recorded their date of birth incorrectly.

‡ This includes individuals who were potentially eligible on the basis of their screening form but who did not enter run-in for a variety of reasons including technical difficulties in processing the form or further information from the patient indicating that they were ineligible or unwilling to take part.

About 40% of all patients who entered the run-in dropped out before randomization. Supplementary Table I gives the reasons for withdrawal: about half had no clinical reason but simply declined to continue. Without this pre-randomization phase, many such withdrawals might instead have occurred early after randomization, resulting in a substantial reduction in statistical power. Towards the end of the 2-month run-in, randomization questionnaires were sent to 22,579 individuals, of whom 15,480 responded that they remained willing and eligible, and were randomized into ASCEND (Figure). Overall 3.7% of those invited were randomized.

### Baseline characteristics of randomized participants

In total 15,480 people (9684 men and 5796 women), average age 63.3 years (SD 9.2) were randomized between June 2005 and July 2011 (Table II). Participants had diabetes (94% type 2) diagnosed a median of about 7 years before randomization. For 16% of participants, their diabetes was managed by diet alone; 25% were using insulin at baseline (with or without other agents); and 58% were using hypoglycemic agents but not insulin. The majority (85%) were overweight or obese at baseline (BMI ≥25 kg/m^2^), and 62% reported taking treatment for hypertension. In those participants for whom baseline blood pressure measurements were available (at the time of blood sampling), the mean systolic blood pressure was 136 mmHg.

**Table II:** Baseline characteristics of study population.

	Male	Female	Total
**Total randomized**	9684 (63%)	5796 (37%)	15,480 (100%)
**Age at randomization (years)**			
<50	582 (6%)	507 (9%)	1089 (7%)
≥50, <60	2888 (30%)	1613 (28%)	4501 (29%)
≥60, <70	3944 (41%)	2303 (40%)	6247 (40%)
≥70	2270 (23%)	1373 (24%)	3643 (24%)
Mean age (SD)	63.3 (9.1)	63.1 (9.4)	63.3 (9.2)
**Body mass index (kg/m^2^)**⁎			
<25	1385 (14%)	864 (15%)	2249 (15%)
≥25, <30	3883 (40%)	1646 (28%)	5529 (36%)
≥30, <35	2666 (28%)	1574 (27%)	4240 (27%)
≥35	1449 (15%)	1512 (26%)	2961 (19%)
Unknown	301 (3%)	200 (3%)	501 (3%)
Mean body mass index (SD)	30.1 (5.6)	31.7 (7.1)	30.7 (6.3)
**Type of diabetes**†			
Type 1	518 (5%)	393 (7%)	911 (6%)
Type 2	9166 (95%)	5403 (93%)	14,569 (94%)
**Diabetes management**			
Diet only	1502 (16%)	1027 (18%)	2529 (16%)
Any hypoglycemic agent but not insulin	5816 (60%)	3204 (55%)	9020 (58%)
Insulin +/− other hypoglycemic agent	2366 (24%)	1565 (27%)	3931 (25%)
**Duration of diabetes (years)**			
<5	2991 (31%)	1900 (33%)	4891 (32%)
≥5, <10	2728 (28%)	1606 (28%)	4334 (28%)
≥10, <20	2287 (24%)	1250 (22%)	3537 (23%)
≥ 20	1150 (12%)	712 (12%)	1862 (12%)
Unknown	528 (5%)	328 (6%)	856 (6%)
Median duration of diabetes (IQR)	7 (3–13)	7 (3–12)	7 (3–13)
**Systolic blood pressure (mmHg)**‡			
<130	1961 (20%)	1433 (25%)	3394 (22%)
≥130, <140	1931 (20%)	1160 (20%)	3091 (20%)
≥140	2965 (31%)	1590 (27%)	4555 (29%)
Unknown	2827 (29%)	1613 (28%)	4440 (29%)
Mean systolic blood pressure (SD)	136.9 (15.2)	134.9 (15.3)	136.2 (15.3)
**Other Factors**§			
Reported treated hypertension (n = 15,368)	5854 (60%)	3679 (63%)	9533 (62%)
Current smoker (n = 15,307)	778 (8%)	501 (9%)	1279 (8%)
Diabetic retinopathy (n = 15,336)	1875 (19%)	1148 (20%)	3023 (20%)
**Ethnic origin**			
White	9331 (96%)	5604 (97%)	14,935 (96%)
Indian/Pakistani/Bangladeshi	141 (1%)	43 (<1%)	184 (1%)
African/Caribbean	79 (<1%)	61 (1%)	140 (<1%)
Other/unknown	133 (1%)	88 (2%)	221 (1%)

⁎ Based on self-reported height and weight

† Based on a broad clinical definition involving age at diagnosis of diabetes, use of insulin within one year of diagnosis and BMI

‡ From blood and urine consent form, generally before randomization

§ Reported by participant on randomization questionnaire.

Blood and urine kits were sent to 22,858 patients who entered the pre-randomization phase and who had not informed the coordinating center that they wished to withdraw before the kits were due to be sent. Samples (either blood or urine or both) were received by the laboratory from 13,270 individuals, among whom 11,685 were subsequently randomized. Samples received from about 1800 participants were not deemed usable as a result of inadequate sample volume, incomplete consent or delays in sample receipt at the central laboratory. Baseline biochemical measures are shown in Table III. Among the 9813 participants with baseline HbA1c available, only 31% achieved target levels for glucose control of <6.5% (48 mmol/mol). By contrast, 4554 participants (46% of those with measures available) had a total cholesterol <4.0 mmol/L. Supplementary Table II provides the baseline characteristics of those participants with a usable baseline blood sample and indicates that they were generally representative of the full study population.

**Table III:** Biochemical measures assessed during pre-randomization run-in phase.

	Male	Female	Total
**Total cholesterol (mmol/L) (n = 9819)**			
<4.0	3175 (52%)	1379 (37%)	4554 (46%)
≥4.0, < 5.0	2193 (36%)	1602 (43%)	3795 (39%)
≥5.0	716 (12%)	754 (20%)	1470 (15%)
Mean (SD)	4.0 (0.8)	4.4 (0.9)	4.2 (0.9)
**HDL cholesterol (mmol/L) (n = 9800)**			
<1.0	1751 (29%)	419 (11%)	2170 (22%)
≥1.0, < 1.5	3466 (57%)	2057 (55%)	5523 (56%)
≥1.5	855 (14%)	1252 (34%)	2107 (22%)
Mean (SD)	1.2 (0.3)	1.4 (0.4)	1.3 (0.4)
**Non-HDL cholesterol (mmol/L) (n = 9800)**			
<2.5	2187 (36%)	1203 (32%)	3390 (35%)
≥2.5, < 3.5	2693 (44%)	1700 (46%)	4393 (45%)
≥3.5	1192 (20%)	825 (22%)	2017 (21%)
Mean (SD)	2.9 (0.8)	3.0 (0.9)	2.9 (0.8)
**Apolipoprotein B (mg/dL) (n = 9779)**			
<70	1900 (31%)	947 (25%)	2847 (29%)
≥70, < 90	2386 (39%)	1516 (41%)	3902 (40%)
≥90	1766 (29%)	1264 (34%)	3030 (31%)
Mean (SD)	80.8 (20)	84.3 (21)	82.1 (21)
**Apolipoprotein A1 (mg/dL) (n = 9799)**			
<130	1781 (29%)	403 (11%)	2184 (22%)
≥130, < 160	3030 (50%)	1669 (45%)	4699 (48%)
≥160	1259 (21%)	1657 (44%)	2916 (30%)
Mean (SD)	143.5 (23)	159.3 (26)	149.5 (25)
**HbA1c DCCT % (IFCC mmol/mol) (n = 9813)**			
<6 (42)	734 (12%)	454 (12%)	1188 (12%)
≥6 (42), < 6.5 (48)	1077 (18%)	744 (20%)	1821 (19%)
≥6.5 (48), < 7 (53)	1317 (22%)	790 (21%)	2107 (21%)
≥7 (53), < 7.5 (58)	1057 (17%)	651 (17%)	1708 (17%)
≥7.5 (58)	1895 (31%)	1094 (29%)	2989 (30%)
Mean (SD)	7.2 (55) (1.2 (13))	7.1 (55) (1.2 (13))	7.2 (55) (1.2 (13))
**eGFR (ml/min/1.73m^2^) (n** **=** **9815)**⁎			
≥90	2966 (49%)	1557 (42%)	4523 (46%)
≥60, < 90	2413 (40%)	1603 (43%)	4016 (41%)
≥45, < 60	490 (8%)	379 (10%)	869 (9%)
≥30, < 45	167 (3%)	155 (4%)	322 (3%)
<30	46 (<1%)	39 (1%)	85 (<1%)
Mean (SD)	86.9 (21)	82.3 (21)	85.2 (21)
**Urinary albumin/creatinine ratio (mg/mmol) (n = 9774)**			
<3	5176 (85%)	3350 (90%)	8526 (87%)
≥3, < 30	764 (13%)	324 (9%)	1088 (11%)
≥30	123 (2%)	37 (<1%)	160 (2%)
Median	0.59	0.51	0.55

HDL, High-density lipoprotein; IFCC, International Federation of Clinical Chemistry

⁎ Calculated from blood cystatin c concentration using the CKD-EPI formula.

Non-study medication use was reported on the randomization form and is shown in Supplementary Table III. Three quarters of participants reported taking a statin, and over a third were previously on aspirin but had no clear clinical indication for it (and they and their GP were agreeable to stopping this in order to take part in ASCEND).

### Post-randomization follow-up

Follow-up of ASCEND participants is scheduled to finish in 2017, by which time there will be a median duration of follow-up of at least 7 years. Follow-up questionnaires are sent 6-monthly. In order to ensure completeness of follow-up, if no reply is received to the initial follow-up questionnaire (paper or emailed request), two reminders are sent to non-responders, followed by a telephone call by coordinating centre staff. In late 2014, the option of completing a follow-up form via the internet was introduced,^24^ and around 15% to 20% of responses are now received online. In addition, electronic information about all deaths and cancers is received periodically from central NHS registries.

## Discussion

There remains continuing clinical uncertainty about whether or not aspirin should be recommended for the primary prevention of cardiovascular events in people with diabetes.^13^ This is reflected in the differing and changing recommendations in cardiovascular prevention guidelines. When ASCEND was designed, the American Diabetes Association recommended aspirin use for primary prevention in people with diabetes with one additional risk factor^28^ but, at that time, the UK and European guidelines were more circumspect.29., 30. More recently, both the 2015 UK National Institute for Health and Care Excellence (NICE) guideline for type 2 diabetes^31^ and the 2016 European Guidelines^32^ have advised not offering antiplatelet therapy for adults with type 2 diabetes but without cardiovascular disease. By contrast, the US Preventive Services Task Force now recommends low-dose aspirin for the primary prevention of cardiovascular disease and colorectal cancer in adults aged 50–59 years who have a 10-year cardiovascular risk of at least 10% and are not considered at increased risk for bleeding, irrespective of their diabetes status.^33^

In addition to ASCEND, three other randomized trials of aspirin therapy in similar intermediate-risk populations are anticipated to announce their results during the next 2–3 years,34., 35., 36. providing substantially more data than is currently available about the value of using aspirin in the primary prevention setting. ASCEND will be responsible for almost half of the available data in diabetes and, in addition, will provide one of the first large-scale prospective tests of aspirin for the prevention of cancer. If aspirin is shown to be effective for cancer chemoprevention then this could significantly alter the balance of benefits and risks for its use in primary prevention.

Given recent data, it is unlikely that ASCEND will show benefits from the omega-3 fatty acid allocation of the magnitude that had been anticipated. Nevertheless, the increase in size and duration of exposure beyond that originally planned means that its ability to detect more modest effects of omega-3 fatty acids has increased. In addition, it should be large enough to determine whether (or not) any particular types of patient benefit to a worthwhile extent from such supplementation.

## Conclusion

The current global epidemic of diabetes makes robust evidence about the effects of low-cost prophylactic interventions especially important. For example, demonstrating that primary prevention with aspirin prevents cardiovascular events or cancers, and that the benefits outweigh the risks of bleeding, would be relevant to some hundreds of millions of people worldwide who are at risk of such events but are currently not taking low-dose aspirin. On the other hand, if the risks of serious bleeding outweigh any benefits then these risks could be avoided by the very large numbers of people who are currently using aspirin for primary prevention.

## Appendix A Supplementary data

Supplementary Table 1: Reasons given for withdrawing from ASCEND during the pre-randomization run-in phase1. Supplementary Table 2: Baseline characteristics of those who returned usable blood samples at randomization. Supplementary Table 3: Non[HYPHEN]study medication use reported on randomization form. Image 1

Supplementary Appendix 1: Laboratory Methods. Supplementary Appendix 2: Members of ASCEND Study Collaborative Group. Supplementary Appendix 3: Data Analysis Plan. Image 2
