# Supplementary material for: ASCEND: A Study of Cardiovascular Events iN Diabetes: Characteristics of a randomized trial of aspirin and of omega-3 fatty acid supplementation in 15,480 people with diabetes
Source: Am Heart J. 2018 Apr;198:135–44. doi: 10.1016/j.ahj.2017.12.006 (PMC5971211; doi:10.1016/j.ahj.2017.12.006)
Supplement: Supplementary file 1 — Supplementary Table 1: Reasons given for withdrawing from ASCEND during the pre-randomization run-in phase1. Supplementary Table 2: Baseline characteristics of those who returned usable blood samples at randomization. Supplementary Table 3: Non[HYPHEN]study medication use reported on randomization form. [file mmc1.rtf]

	

A Study of Cardiovascular Events iN Diabetes (ASCEND)	

	
Baseline paper tables – Supplementary Tables Only	

	
	


	


Date freeze date	2017-05-17T11:47:43	
Report generated	2017-07-11T09:08:36	


Reason for withdrawal	Number of participants	
			
GP or practice unwilling to participate	98	(<1%)	
Medical advice*	556	(5%)	
Side effects of study medication	419	(4%)	
    Gastrointestinal side effects	259		
    Increased bleeding or bruising	10		
    Other side effects	150		
Difficulty getting tablets out of blister	14	(<1%)	
Difficulty swallowing tablets or capsule	201	(2%)	
Not eligible†	1453	(13%)	
    Aspirin or other contraindicated drug prescribed by doctor	1039		
    Not compliant with run-in medication	141		
    Ongoing serious medical problem	89		
    Stomach or duodenal ulcer, or gastrointestinal bleeding in last 6 months	53		
    Coronary artery disease	53		
    Cancer	50		
    Transient ischaemic attack or stroke	48		
    Other arterial surgery or planned in next 6 months	14		
Unwilling to continue	5345	(49%)	
Randomization questionnaire not received after 20 weeks	2832	(26%)	
Questionnaire incomplete and unable to contact invitee	49	(<1%)	
Died during run-in	15	(<1%)	
			
Total entering run-in but not randomized	10982	(100%)	
*Includes individuals for whom the GP or other clinician considered aspirin to be contra-indicated; †More than one reason can apply for each participant	

	Total	Usable sample	No usable sample		p	
Total randomized	15480		9821	(63%)	5659	(37%)			
									
Age (years)									
<50	1089		618	(6%)	471	(8%)			
≥50, <60	4501		2528	(26%)	1973	(35%)			
≥60, <70	6247		4074	(41%)	2173	(38%)			
≥70	3643		2601	(26%)	1042	(18%)			
								<0.001	
Mean age	63.3	(9.2)	64.1	(9.1)	61.8	(9.1)		<0.001	
									
Sex									
Male	9684		6085	(62%)	3599	(64%)			
Female	5796		3736	(38%)	2060	(36%)			
								0.042	
									
Current smoker									
Yes	1279		738	(8%)	541	(10%)			
No	14028		8959	(91%)	5069	(90%)			
Unknown	173		124	(1%)	49	(1%)			
								<0.001	
									
Body mass index (kg/m2) (n=14979)*	30.7	(6.3)	30.6	(6.2)	31.0	(6.4)		<0.001	
									
Ethnic origin									
White	14935		9510	(97%)	5425	(96%)			
Indian/Pakistani/Bangladeshi	184		99	(1%)	85	(2%)			
African/Caribbean	140		88	(1%)	52	(1%)			
Other/unknown	221		124	(1%)	97	(2%)			
								0.005	
									
Townsend deprivation index†									
<-3	5104		3237	(33%)	1867	(33%)			
≥-3, <0	6022		3914	(40%)	2108	(37%)			
≥0, <2	2037		1269	(13%)	768	(14%)			
≥2, <4	1315		807	(8%)	508	(9%)			
≥4, <6	703		423	(4%)	280	(5%)			
≥6	261		153	(2%)	108	(2%)			
								0.008	
									
Duration of diabetes (years) (n=14624)	7	(3-13)	7	(3-13)	7	(3-12)		0.139	
									
Type of diabetes									
Type 1	911		550	(6%)	361	(6%)			
Type 2	14569		9271	(94%)	5298	(94%)			
								0.047	
									
Diabetes management									
Diet only	2529		1651	(17%)	878	(16%)			
Any hypoglycaemic agent but not insulin	9020		5682	(58%)	3338	(59%)			
Insulin +/- oral hypoglycaemic agent	3931		2488	(25%)	1443	(25%)			
								0.105	
									
Diabetic retinopathy (self reported)									
Yes	3023		1900	(19%)	1123	(20%)			
No	12313		7830	(80%)	4483	(79%)			
Unknown	144		91	(1%)	53	(1%)			
								0.749	
Self reported hypertension									
Yes	9533		6058	(62%)	3475	(61%)			
No	5835		3697	(38%)	2138	(38%)			
Unknown	112		66	(1%)	46	(1%)			
								0.592	
									
Non-study medication use reported on randomization form									
Statin									
Yes	11653		7422	(76%)	4231	(75%)			
No	3827		2399	(24%)	1428	(25%)			
								0.262	
ACE Inhibitor or ARB use									
Yes	9055		5774	(59%)	3281	(58%)			
No	6425		4047	(41%)	2378	(42%)			
								0.322	
Aspirin (at screening)									
Yes	5508		3572	(36%)	1936	(34%)			
No	9972		6249	(64%)	3723	(66%)			
								0.007	
ACE, Angiotensin converting enzyme; ARB, Angiotensin II receptor blocker; Usable sample defined as a sample of sufficient volume returned to the lab 1-4 days after sample collection, excluding those who did not confirm that they had read and understood the blood and urine sampling patient information sheet, and those whose sample was taken more than 30 days after randomization; Data are presented as mean(SD) for age and body mass index, median(interquartile range) for duration of diabetes; *Calculated from self-reported height and weight; †Calculated using postcode at randomization. A higher value means greater deprivation	

Non-study treatment	Male	Female	Total	
Insulin	2366	(24%)	1565	(27%)	3931	(25%)	
Metformin	6446	(67%)	3647	(63%)	10093	(65%)	
Sulphonylurea	2831	(29%)	1314	(23%)	4145	(27%)	
Thiazolidinedione	1201	(12%)	590	(10%)	1791	(12%)	
Other hypoglycaemic agent	423	(4%)	270	(5%)	693	(4%)	
ACE Inhibitor or ARB	5772	(60%)	3283	(57%)	9055	(58%)	
Beta-blocker	1165	(12%)	864	(15%)	2029	(13%)	
Calcium channel blocker	2535	(26%)	1238	(21%)	3773	(24%)	
Thiazide or related diuretic	1656	(17%)	1301	(22%)	2957	(19%)	
Statin	7300	(75%)	4353	(75%)	11653	(75%)	
NSAID	734	(8%)	599	(10%)	1333	(9%)	
Proton pump inhibitor	1231	(13%)	1023	(18%)	2254	(15%)	
Aspirin (at screening)	3697	(38%)	1811	(31%)	5508	(36%)	
ACE, Angiotensin converting enzyme; ARB, Angiotensin II receptor blocker; NSAID, Non-steroidal anti-inflammatory drug	
