# Supplementary material for: ASCEND: A Study of Cardiovascular Events iN Diabetes: Characteristics of a randomized trial of aspirin and of omega-3 fatty acid supplementation in 15,480 people with diabetes
Source: Am Heart J. 2018 Apr;198:135–44. doi: 10.1016/j.ahj.2017.12.006 (PMC5971211; doi:10.1016/j.ahj.2017.12.006)
Supplement: Supplementary file 2 — Supplementary Appendix 1: Laboratory Methods. Supplementary Appendix 2: Members of ASCEND Study Collaborative Group. Supplementary Appendix 3: Data Analysis Plan. [file mmc2.pdf]

Supplementary Appendix 1:

Laboratory Methods

Blood levels of total cholesterol, apolipoprotein-B and apolipoprotein-A1, and urinary creatinine and albumin were assayed using Beckman-Coulter CX4, LX20 and DxC800 clinical chemistry analysers and manufacturers' reagents, calibrators and settings (Beckman-Coulter, UK). HDL cholesterol was assayed using the Wako HDL method (antibodies bind LDL, VLDL and chylomicrons so that only the HDL is measured). HbA1c analysis was performed by HPLC using EDTA whole blood on an Arkray HA8160 analyser and reagents with a calibrator supplied by Menarini Diagnostics UK traceable to IFCC and DCCT reference standards. Cystatin C was measured on the samples collected at baseline by particle enhanced Immunonephelometry using a Dade Behring BNII Nephelometer using manufacturers' reagents, calibrators and settings (Dade Behring/Siemens Germany). In order to allow comparison of the eGFR values calculated from these cystatin C measurements with other studies, frozen plasma samples from 300 randomly selected participants were reanalysed using the Siemens BN Prospec Nephelometer, Dade Behring/Siemens Germany which is aligned to the International Federation of Clinical Chemistry and Laboratory Medicine reference materials developed following the standardisation of cystatin C measurement procedures in 2010.[1] The resulting regression equation (corrected cystatin C = 0.0816 + 0.9782 x measured cystatin C value) was used to adjust the baseline cystatin C values to align them with the IFCC traceable method. Estimated GFR was calculated using the CKD-epi formula.[2]

References

1. Grubb A, Blirup-Jensen S, Lindstrom V, Schmidt C, Althaus H, Zegers I. First certified reference material for cystatin C in human serum ERM-DA471/IFCC. Clinical chemistry and laboratory medicine. 2010;48(11):1619-21. Epub 2010/11/03.
2. Inker LA, Schmid CH, Tighiouart H, Eckfeldt JH, Feldman HI, Greene T, Kusek JW, Manzi J, Van Lente F, Zhang YL, Coresh J, Levey AS. Estimating glomerular filtration rate from serum creatinine and cystatin C. N Engl J Med. 2012;367(1):20-9. Epub 2012/07/06.

Supplementary Appendix 2:

Members of ASCEND Study Collaborative Group

Steering Committee

Chairman: R Collins, *Study coordinator:* J Armitage; *Clinical coordinator:* L Bowman; *Statisticians:* S Parish, R Peto; *Administrative coordinator:* J Barton; *Lay member:* D Simpson; *Other members:* A Adler, T Aung, C Baigent, HJ Bodansky, A Farmer, R Haynes, R McPherson, M Mafham, HAW Neil, N Samani, P Sleight, P Weissberg.

Data Monitoring Committee

Chair: P Sandercock, *Members:* H Gerstein, R Gray, C Hennekens.

Coordinating Office (Clinical Trial Service Unit, Nuffield Department of Population Health, University of Oxford):

Administration and support: J Barton, L Fletcher, K Murphy (coordinators); P Achiri, A Armitage, S Bateman, V Booker, K Brown, F Butcher, E Butler, S Butler, L Cobb, L Cobb, A Collett, P Colmenero, J Crowther, S Fathers, K Frederick, E Goodfellow, E Goodwin, C Hope, A Karnad, V Keyte, M King, S Knight, N Lam, R Lee, O Machin, N Mohammed, A Panicker, L Pank, A Papadaki, E Pearson-Burton, S Pickworth, YR Qiao, A Radley, E Roberts, K Roby, J Sayer, S Shah, S Smith, S Sutherland, H Thorne, A Timadjer, K Vandenberg, M Willett, M Wincott, C Wise, J Woods, S Yates.

Clinical support and adjudication: A Alexander, J Armitage, T Aung, S Beebe, J Black, L Bowman, L Bromhall, R Bulbulia, F Chen, H Cowan, T Dasgupta, K Gaba, R Haynes, W Herrington, L Hirt, A Isaew, J James, P Judge, A Lawson, D Lewis, R Llewellyn-Bennett, H Lochhead, M Mafham, W Majoni, C Murray, K Naessens, T Porter, K Rahimi, C Reith, E Sammons, B Storey, M Taylor-Clarke, V Toghill, J Tomson, K Walter, E Waters, L Young.

Statistics and computing: P Harding, M Lay, S Parish (coordinators); L Adamska, D Bennett, C Berry, J Booth, Y Bu, G Buck, G Coates, J Cox, M Craig, J Crawford, P Dalton, C Daniels, C Dawe, A Delmestri, X Guo, J Karreman, R Kurien, A McDougall, Y Mostefai, A Murawska, M Nunn, N Prajapati, J Price, W Stevens, S Syed, M Turakani, K Wallendszus, A Young.

CTSU Wolfson Laboratory: S Clark, K Emmens, M Hill, K Kourellias, M Radley, J Wintour (coordinators); M Allworth, L Boggs, T Chavagnon, R Cox, J Cwikowska, T Glass, N Goodwin, A Gordon, J Gordon, C Guest, C Hickman, J Hill, R Hrusecka, N Illingworth, JM Ji, M Lacey, N Luker, K Nafousi, S Norris, N Plunkett, L Sansom, R Shellard, J Taylor, P Taylor, J Wheeler, T Williams, M Yeung.

Collaborators

Addenbrooke's Hospital, Cambridge: A Adler; *Ashford Hospital, Ashford:* U Meyer-Bothling; *NHS Bolton, Bolton:* J Dean; *Charing Cross Hospital, London:* J Car, A Dornhorst; *Chesterfield Royal Hospital, Chesterfield:* R MacInerney; *City Hospital, Birmingham, Birmingham:* P De; *Colchester General Hospital, Colchester:* C Bodmer; *NHS Cumbria, Carlisle:* R Wagstaff; *NHS Derby City, Derby:* M Browne; *NHS Derbyshire County, Chesterfield:* T Humphries; *Dewsbury & District Hospital, Dewsbury:* T Kemp; *Frimley Park Hospital, Frimley:* G Menon; *Gloucester Royal Hospital, Gloucester:* T Ulahannan; *Harrogate District Hospital, Harrogate:* P Hammond; *Hemel Hempstead General Hospital, Hemel Hempstead:* C Johnston; *Hospital of St Cross, Rugby:* JP O'Hare; *Huddersfield Royal Infirmary, Huddersfield:* T Burrows, H Griffiths; *Hull Royal Infirmary, Hull:* S Atkin, C Walton; *Ipswich Hospital, Ipswich:* P Twomey; *James Cook University Hospital, Middlesbrough:* R Bilous; *Kidderminster Hospital, Kidderminster:* P Newrick; *King's Mill Hospital, Sutton-in-Ashfield:* I Idris, R Lloyd-Mostyn; *Lavender Hill Group Practice, London:* J Gray; *Leeds General Infirmary, Leeds:* HJ Bodansky; *Leighton Hospital, Crewe:* S Mallya; *Lincoln County Hospital, Lincoln:* K Sands; *Macclesfield District General Hospital, Macclesfield:* Z Hasan; *Manchester Royal Infirmary, Manchester:* R Malik; *Newcastle General Hospital, Newcastle-upon-Tyne:* G Hawthorne, C Jones-Unwin; *Newham University Hospital, London:* S Gelding; *NIHR Clinical Research Network, Keele:* M Porcheret; *North Tyneside General Hospital, North Shields:* S Bennett; *Northumbria Healthcare, Ashington:* N Lewis-Barned; *Peninsula Medical School (Primary Care), Exeter:* P Evans; *Peterborough City Hospital, Peterborough:* S Martin; *Pilgrim Hospital, Boston:* C Nyman, S Olczak; *Pontefract General Infirmary, Pontefract:* C White; *Poole General Hospital, Poole:* A McLeod; *Princess Royal Hospital, Telford:* N Capps; *Queen Alexandra Hospital, Portsmouth:* M Cummings; *Queen's Hospital, Burton-on-Trent:* T Reynolds; *Queen's Medical Centre, Nottingham:* T Gazis; *Rotherham District General Hospital, Rotherham:* R Muthusamy, S Muzulu; *Royal Albert Edward Infirmary Wrightington, Wigan:* S Natha; *Royal Berkshire Hospital, Reading:* H Simpson; *Royal Blackburn Hospital, Blackburn:* S Ramtoola; *Royal Bolton Hospital, Bolton:* A Hutchesson; *Royal Cornwall Hospital, Truro:* S Fleming; *Royal Derby Hospital, Derby:* GD Tan; *Royal Devon & Exeter Hospital, Exeter:* K MacLeod (deceased); *Royal Hallamshire Hospital, Sheffield:* C Brand; *Royal Liverpool University Hospital, Liverpool:* D Broadbent, J Vora; *Royal Oldham Hospital, Oldham:* D Bhatnagar, B Mishra; *Royal United Hospital Bath, Bath:* J Reckless; *Sandwell District General Hospital, West Bromwich:* E Hughes, D Robertson; *South Tyneside District Hospital, South Shields:* C Thomas; *St Helier Hospital, Carshalton:* S Hyer, A Rodin; *St James's University Hospital, Leeds:* S Gilbey; *St Mary's Hospital, London Paddington:* D Gable; *St Peter's Hospital, Chertsey:* M Baxter; *Stepping Hill Hospital, Stockport:* P Hale, N Kong, G Burrows; *The Royal London Hospital, London:* T Chowdhury, D Peterson; *Torbay Hospital, Torquay:* R Paisey; *University Hospital of Wales, Cardiff:* R McPherson; *Warrington Hospital, Warrington:* P Chattington; *Watford General Hospital, Watford:* M Clements; *Weston General Hospital, Weston-super-Mare:* P Singhal; *William Harvey Hospital, Ashford:* A Jafree, C Williams; *Worthing Hospital, Worthing:* G Caldwell, M Signy; *Wycombe General Hospital, High Wycombe:* I Gallen; *York District Hospital, York:* P Jennings.

Supplementary Appendix 3:

ASCEND: A Study of Cardiovascular Events in Diabetes

A randomized trial of aspirin and omega-3 fatty acids for the prevention of cardiovascular disease and cancer in diabetes.

Data Analysis Plan

Table of Contents

Table of Contents 5
1.0 Background 6
  1.1 Cardiovascular disease 6
  1.2 Cancer 7
  1.3 Major bleeding 7
2.0 Purpose of this Data Analysis Plan 8
3.0 Comparisons during the scheduled treatment period 8
  3.1 Efficacy assessments 8
  3.2 Safety assessments 9
  3.3 Further exploratory assessments 10
  3.4 Analyses of biochemical data 10
4.0 Comparisons during long-term follow-up 10
  4.1 Primary long-term assessment 11
  4.2 Secondary long-term assessments 11
  4.3 Further long-term exploratory assessments 11
5.0 Details of analyses 11
  5.1 Methods of analysis 11
  5.2 Allowance for multiplicity of comparisons 12
  5.3 Tests for heterogeneity and trend in effects 12
  5.4 Imputation of missing data 13
  5.5 Study average compliance analyses 13
6.0 References 14
Appendix 1: Definition of ASCEND event terms 16
Appendix 2: Derivation of vascular risk score 20
Appendix 3: Censoring rules for analysis of events during the scheduled treatment period 21

1.0 Background

ASCEND is a randomized trial which aims to determine whether 100mg daily aspirin and/or supplementation with 1g capsules containing 90% omega-3 fatty acids (FA: 0.41g eicosapentaenoic acid [EPA], 0.34g docosahexaenoic acid [DHA]) daily prevents cardiovascular events in patients with diabetes who do not already have clinically manifest arterial disease (without leading to significant bleeding or other adverse events). It will also assess whether aspirin prevents cancer. The study design is a 2x2 factorial placebo-controlled randomized trial, in which participants are randomly allocated to aspirin or placebo, and separately to omega-3 FA or placebo.

Between 2005 and 2011, 423,403 people with diabetes from around the UK were sent a postal invitation to take part. Of those, 26,462 eligible patients entered a 2-month pre-randomization run-in, during which they received placebo aspirin and placebo omega-3 FA, and 15,480 (who were still willing and eligible to participate at the end of run-in) were randomized.[3] Follow-up during the treatment period is predominantly mail-based, but with additional information about death, cancers and hospital admissions from central registries.

After the end of the scheduled treatment period, information about causes of death and incident cancers, along with Hospital Episode Statistics (HES) data will continue to be collected from NHS Digital (previously the Health and Social Care Information Centre (HSCIC)) on all surviving ASCEND participants (unless they have withdrawn consent). These data will be used to assess whether any benefits of aspirin observed within the trial continue long-term or emerge during longer-term follow-up.

1.1 Cardiovascular disease

Initially, a sample size of at least 10,000 randomized patients treated for about 5 years was proposed. This calculation was based on an expected rate of 2% in the control groups (based on previous trials in similar diabetic populations) for the composite outcome of non-fatal myocardial infarction, non-fatal stroke or cardiovascular death (excluding confirmed cerebral haemorrhage) and a proportional reduction of 15-20% in the active arms (aspirin and omega-3 FA have reduced risk of cardiovascular events by around 25%[4] and 15-20%[5] respectively in high-risk populations). However, it was pre-specified that the Steering Committee could decide to stop the study early in the light of recommendations from the independent Data Monitoring Committee, or increase the sample size or prolong the scheduled treatment period if the blinded event rates (i.e. active and placebo groups combined) proved to be substantially lower than anticipated.

In 2010, based on blinded analyses among the first few thousand randomized participants, the blinded reported rate overall (i.e. in both treatment groups combined) of the composite outcome was lower than expected: approximately 0.6% per annum, rather than the 2% rate used in the original statistical power calculations. In addition, the Anti-thrombotic Treatment Trialists' Collaboration meta-analysis[6] of primary prevention trials of aspirin reported that the proportional reduction in the

composite outcome attributable to aspirin was more likely to be in the range 12-15%, rather than the 20-25% range used in the original power calculations. In light of these factors, in 2010 while recruitment was still ongoing, ethics committee approval was obtained to increase the sample size to at least 15,000 participants, to extend the duration of follow-up to at least 7 years, and to extend the primary endpoint to include transient ischaemic attacks (TIAs). The blinded annual event rate for this wider endpoint including TIAs is currently about 1.2-1.3%, and the trial has 89% power at 2p<0.05 after 7.5 years of follow-up to detect a 15% proportional reduction in the primary endpoint (i.e. 15 SVEs or TIAs avoided per 1000 patients treated for 7.5 years).

1.2 Cancer

Based on post-hoc analyses of long-term follow-up data from selected randomized trials of aspirin and of observational studies of cancer incidence in relation to aspirin use, it has been suggested that aspirin protects against various forms of cancer (particularly gastrointestinal [GI] cancers), but that the effect may take some years to emerge.[7-15] For example, 20 year follow-up of 4 trials of low dose aspirin versus control involving 14,000 patients showed a 24% (95% CI 4-40%; p=0.02) reduction in the risk of colorectal cancer incidence based on about 400 events.[8] If this benefit is confirmed in prospective randomized trials, it could have important implications for the balance of benefits and risks of aspirin in primary prevention.

ASCEND provides one of the first opportunities to test this hypothesis prospectively in a large-scale long-term randomized trial, both during the 7-year treatment phase and during prolonged post-trial follow-up.

For the in-trial analysis, the primary cancer endpoint is any GI tract cancer. Little or no treatment effect is expected before about 3 years (based on previously published trial data[10]), therefore limiting the statistical power to detect plausible effects of aspirin during the scheduled treatment period. For example, with an expected 430 GI tract cancers during the treatment period, of which about 250 would occur after 3 years, there would only be 60% power at 2p<0.05 to detect a 30% proportional risk reduction. Pre-specified analyses by period of follow-up will assess whether effects are increasing with time from randomization. However, the main focus of the analyses will be after longer-term post-trial follow-up, when there will be much better power to detect plausible differences in cancer incidence between the arms due to larger numbers of events. For example, about 5 years after the end of the scheduled treatment period, there would be >90% and 76% power respectively to detect 25% and 20% proportional reductions in the incidence of any GI cancer tract at 2p<0.05 (ie preventing 10-12 GI tract cancers per 1000 over 12.5 years including 7.5 years of treatment).

1.3 Major bleeding

Aspirin is known to increase the risk of major bleeding (both intracranial and extra-cranial), with meta-analyses of randomized trials[6] suggesting an increase of about

half (e.g. RR 1.54 [1.30-1.82] for any extra-cranial bleed) and with the risk factors for bleeding appearing to be similar to the risk factors for vascular events. Based on the current blinded rate of major bleeding of 0.5-0.6% per annum in ASCEND, it has 90% power at 2p <0.01 to detect an increase in bleeding with aspirin of at least 35% (ie 13 extra bleeds per 1000 patients treated for 7.5 years).

Hence, ASCEND should be able to assess reliably the balance of plausible increases in the incidence of major bleeds versus plausible reductions in the incidence of occlusive vascular events and cancers with the prophylactic use of low-dose aspirin in primary prevention. In addition, by using a factorial design, it should be able to provide reliable information about the effects of using omega-3FA in primary prevention.

2.0 Purpose of this Data Analysis Plan

The purpose of this Data Analysis Plan is to describe the strategy, rationale and statistical methods which will guide assessment of the main analyses of efficacy and safety for aspirin and for omega-3 FA in the ASCEND trial. The nature of all potential subsidiary analyses and the content of subsequent publications cannot be pre-specified, but the general approach to exploratory analyses is outlined. Analyses and reports will be prepared by the coordinating centre in the Clinical Trial Service Unit, University of Oxford.

3.0 Comparisons during the scheduled treatment period

All comparisons will involve comparing outcomes during the scheduled treatment period (i.e. from date of randomization to date of final follow-up regardless of whether the participant continues on study treatment or not) among all those participants allocated at randomization to receive aspirin (or, respectively, omega-3 FA) daily versus all those allocated to receive matching placebo (i.e. “intention-to-treat” analyses). Unless otherwise indicated, analyses will be of the first occurrence of the specified outcome. For those events that have been subject to adjudication (see Protocol), analyses include all confirmed and unrefuted events.

3.1 Efficacy assessments

The primary efficacy assessments will involve intention-to-treat comparisons among all randomized participants of allocation to aspirin versus placebo and, separately, of omega-3 FA versus placebo on the first occurrence of any “**serious vascular event” (SVE)**, defined as:

- non-fatal myocardial infarction; or
- non-fatal stroke (excluding confirmed intracranial haemorrhage) or TIA; or
- vascular death excluding confirmed intracranial haemorrhage (defined as International Classification of Diseases 10th revision [ICD-10] I00-52 or I63-99, ie, excluding subarachnoid haemorrhage [I60], intracerebral haemorrhage [I61], and other non-traumatic intracranial haemorrhage [I62]).

Secondary efficacy assessments of aspirin will involve intention-to-treat comparisons during the scheduled treatment period among all randomized participants on the first occurrence of:

i. any incident gastrointestinal (GI) tract cancer (i.e. any GI cancer excluding pancreas and hepatobiliary), overall and after exclusion of the first three years of follow-up;
ii. the expanded vascular endpoint of "SVE or revascularisation" (including coronary and non-coronary revascularisations).

Secondary assessments of the efficacy of omega-3 fatty acids will involve intention-to-treat comparisons during the scheduled treatment period among all randomized participants on the first occurrence of the expanded vascular endpoint of "SVE or revascularisation".

3.2 Safety assessments

It is expected that aspirin will increase the risk of a range of bleeding outcomes, but there are not any pre-specified safety outcomes for omega-3 FA.

The primary safety assessments will involve intention-to-treat comparisons among all randomized patients of allocation to aspirin versus placebo on the first occurrence of "any major bleed", defined as:

- any confirmed intracranial haemorrhage (including intracerebral, subarachnoid, subdural or any other intracranial haemorrhage); or
- sight-threatening eye bleeding; or
- any other serious[†] bleeding episode.

Secondary safety assessments of aspirin will involve intention-to-treat comparisons during the scheduled treatment period among all randomized participants on the first occurrence of:

i. haemorrhagic stroke (i.e. intracerebral or subarachnoid haemorrhage), overall and by level of disability (fatal; disabling; non-disabling; unknown disability);
ii. any major bleed by site:
    a. intracranial haemorrhage and separately its components (intracerebral, sub-arachnoid, subdural and other haemorrhage);
    b. sight-threatening eye bleed;
    c. serious gastrointestinal (GI) haemorrhage;
    d. other serious bleed (ie any extra-cranial, extra-ocular or non GI haemorrhage).

[†] Bleeding which required hospitalisation or transfusion, or is fatal or disabling (Appendix 1)

3.3 Further exploratory assessments

Further exploratory assessments will be made of other possible beneficial or adverse effects of aspirin and of omega-3 FA during the scheduled treatment period. For efficacy, these will include total and cause-specific mortality, other vascular outcomes (eg, any coronary heart disease, components of SVE and SVE excluding TIA), total and site-specific cancer, and microvascular complications and, for omega-3 FA, non-fatal or fatal arrhythmias. For safety, these include all bleeding adverse events (including both serious and non-serious events) overall and by degree of severity. (See Appendix 1 for details of efficacy and safety categories.)

In addition, while it is not anticipated that the proportional effects of aspirin or omega-3 FA on particular outcomes will vary depending on particular baseline characteristics, subgroup analyses of the expanded endpoint of “SVE or revascularisation”, of GI tract cancer incidence and of any major bleed will be done for certain key prognostic variables, including sex, age at randomization (<60; ≥60 <70; ≥70 years), duration of diabetes (<9; ≥9 years), use of aspirin prior to randomization and vascular risk score (predicted 5-year risk of SVE without aspirin or omega-3 FA <5%, ≥5% to <10%, or ≥10% - see Appendix 2 for derivation).Total cancer incidence will be explored by time since randomisation (<3; ≥3, 5; ≥5 years).

Changes in body weight, and comparisons of the prevalence of use of cardiovascular and other relevant drugs at the end of the study treatment period, will also be assessed (see Appendix 1). Sub-studies (to be described separately) will assess the effects of study treatments on retinopathy and cognitive function.

3.4 Analyses of biochemical data

During the 2-month placebo “run-in” phase, blood and urine samples were collected locally in GP surgeries and mailed to the central CTSU laboratory. Samples were obtained from around 75% of randomized participants to measure baseline lipids (total and HDL cholesterol, and apolipoproteins B and A1), HbA1c and renal function (cystatin C and urinary albumin:creatinine ratio).[16] In addition, blood pressure was measured at the time of sample collection and reported to the ASCEND team. Blood and urine samples were also collected during follow-up in a random sample of ASCEND participants (~10%) at median follow up of approximately 2.5 years for analysis of the same analytes by treatment allocation. To assess the biochemical effect of study treatment allocation urinary 11-dehydro thromboxane B2, as a marker of aspirin effectiveness, and red cell omega-3 FA index were assayed in a subgroup of patients with both blood and urine samples available during run-in and follow-up.

4.0 Comparisons during long-term follow-up

Analyses are planned for 5 and 10 years after the end of the scheduled treatment period (ie including data up to 31.3.2022 and 31.3.2027), and will involve intention-to-treat comparisons among all those previously allocated aspirin compared with those previously allocated placebo.

4.1 Primary long-term assessment

The primary long-term efficacy assessment of aspirin will involve intention-to-treat comparisons among all randomized participants of the original allocation to aspirin versus placebo on the first occurrence of any incident gastrointestinal tract cancer.

4.2 Secondary long-term assessments

Secondary long-term efficacy assessments of aspirin will involve intention-to-treat comparisons among all randomized participants of the original allocation to aspirin versus placebo on the first occurrence of:

i. Any cancer (excluding non-fatal non-melanoma skin cancer);
ii. Colorectal cancer;
iii. Death from cancer;
iv. Incident GI tract cancer by time since randomization: <3; ≥3 <5; ≥5 <10; ≥10 <20; ≥20 years.

4.3 Further long-term exploratory assessments

Further exploratory assessments will be made of the long-term effects of the original allocation to aspirin versus placebo on other site-specific cancers (see Appendix 1) and colorectal cancer subdivided into proximal and distal colon. In addition, assessment will be made of any cancer (excluding non-fatal non-melanoma skin cancer) excluding cancers diagnosed within 3 years after randomization; cancer with metastatic spread at the time of diagnosis; adenocarcinoma and non-adenocarcinoma solid tumours overall, and by the presence of metastatic spread. While it is not anticipated that the proportional effects of the long-term effects of aspirin will vary depending on particular baseline characteristics, analyses of primary and secondary outcomes will be undertaken by certain key prognostic variables described in Section 3.3.

Exploratory assessments will also be made of the long-term effects of the original allocation to aspirin or placebo on SVEs, hospitalisation or death from heart failure, and dementia.

5.0 Details of analyses

5.1 Methods of analysis

Pre-specified assessments will involve “intention-to-treat” analyses among all randomized patients of allocation to aspirin versus allocation to placebo tablets and, separately, of omega-3 fatty acids or placebo (irrespective of compliance). Use of a factorial design instead of a simple 2-way design is anticipated to have little or no effect on the statistical sensitivity with which the effects of aspirin can be assessed (or vice versa for omega-3 FA).[17, 18] The assessments of effects of aspirin will, therefore, be made without stratification by omega-3 FA allocation or other factors (and, similarly, for assessments of effects of omega-3 FA).

All time-to-event analyses will be based on the first relevant unrefuted event: that is, an event of a particular type will be included in an analysis provided that it has either been confirmed or has not been refuted during the adjudication process. For assessments during the scheduled treatment period, events will be censored at the date defined in Appendix 3. The "logrank" test[18, 19] will be used to calculate average event rate ratios, confidence intervals, and two-sided p-values. When event rate ratios are <0.5 or >2, logrank event rate ratios will be supplemented by hazard ratios calculated by Cox regression.[20, 21] For comparisons of the overall proportions of affected individuals, irrespective of time, standard Mantel-Haenszel methods for analysis of contingency tables[22] and/or standard regression methods, will be used. Comparisons of biochemical measures (transformed if necessary) between those allocated active treatment versus those allocated placebo will use standard linear regression, adjusted for baseline values when available (i.e. analysis of covariance).

5.2 Allowance for multiplicity of comparisons

No allowance will be made for multiple hypothesis testing for the primary assessments for the in-trial and long-term follow-up, and results will be considered statistically significant if the 2-sided p-value is <0.05. For the secondary and exploratory assessments, allowance in their interpretation will be made for multiple hypothesis testing,[17, 18] taking into account the nature of events (including timing, duration and severity) and evidence from other studies. The larger the number of events on which a comparison is based and the more extreme the p-value (or, analogously, the further the lower/upper confidence limit for the absolute treatment effect is from zero) after allowance has been made for the nature of the particular comparison (i.e. primary, secondary, pre-specified or exploratory), the more reliable the comparison and, hence, the more definite a finding will be considered. Analyses of fatal events will be interpreted in the light of observed effects on relevant non-fatal events.

5.3 Tests for heterogeneity and trend in effects

The limited number of vascular events expected in this study makes direct assessment of the effects of treatment in some subcategories of patient or of vascular events (e.g. fatal versus non-fatal) unreliable. Chance alone may lead to there being no apparent effect in several small subgroups in which the effect of treatment really is about the same as is observed overall. In such circumstances, "lack of direct evidence of benefit" is not good "evidence of lack of benefit", and clearly significant overall results would provide strong indirect evidence of benefit in some small subgroups where the results, considered in isolation, are not conventionally significant (or, even, perhaps, slightly adverse).[17, 18, 23] Hence, unless the proportional effect in some specific subcategory is clearly different from that observed overall, the effect in that subcategory is likely to be best estimated indirectly by applying the proportional effect observed among all patients in the trial

to the absolute risk of the event observed among control patients in that category.[23]

Tests for heterogeneity of the proportional effect on particular outcomes in specific subgroups will be used (with allowance for multiple comparisons and for other differences between the subgroups) to determine whether the effects in those subgroups are clearly different from the overall effect.[24] If, however, such subgroups can be arranged in some meaningful order (e.g. duration of diabetes) then assessment of any trend in the proportional effects on outcome will also be made.

5.4 Imputation of missing data

For the assessment of the effect of treatment by subgroups, missing data will be handled differently depending on the proportion missing. Where there are missing subgroup data (e.g. systolic blood pressure) for a substantial proportion of patients, they will be presented in a separate "unknown" category. When only a small proportion is missing, they will not contribute to the subgroups, but will contribute to the overall result.

5.5 Study average compliance analyses

Participants at risk of the event in question are considered compliant if a follow-up form was received that indicates the participant took their treatment "every day" or "most days" during the time period considered. Participants are also considered compliant if they had previously been compliant, were still receiving medication and had not reported stopping treatment, and information was received within the previous 7 months.

Although analyses will be by intention-to-treat, non-compliance with the allocated treatments will tend to underestimate the effect produced by full compliance. The effect of full compliance with aspirin will be estimated based on the observed intention-to-treat effects on the primary and secondary outcomes and average in-trial compliance with the randomized treatment (determined by participant reports and treatment issue records). For example, if study average compliance is 70% in the aspirin-allocated group and 10% of the placebo-allocated group took active aspirin then the difference in the proportion taking the active treatment would be 60%. If the proportional reduction in the primary endpoint estimated from intention-to-treat analysis is 10% at 5 years then this suggests that full compliance with the treatment would produce a proportional reduction in risk nearer to 17% (i.e. 10 x 100/60).

6.0 References

1. Grubb A, Blirup-Jensen S, Lindstrom V, Schmidt C, Althaus H, Zegers I. First certified reference material for cystatin C in human serum ERM-DA471/IFCC. Clinical chemistry and laboratory medicine. 2010;48:1619-21.
2. Inker LA, Schmid CH, Tighiouart H, Eckfeldt JH, Feldman HI, Greene T, et al. Estimating glomerular filtration rate from serum creatinine and cystatin C. N Engl J Med. 2012;367:20-9.
3. Aung T, Haynes R, Barton J, Cox J, Murawska A, Murphy K, et al. Cost-effective recruitment methods for a large randomised trial in people with diabetes: A Study of Cardiovascular Events iN Diabetes (ASCEND). Trials. 2016;17:286.
4. Antiplatelet Trialists' Collaboration. Collaborative overview of randomised trials of antiplatelet therapy--I: Prevention of death, myocardial infarction, and stroke by prolonged antiplatelet therapy in various categories of patients. BMJ. 1994;308:81-106.
5. Bucher HC, Hengstler P, Schindler C, Meier G. N-3 polyunsaturated fatty acids in coronary heart disease: a meta-analysis of randomized controlled trials. The American journal of medicine. 2002;112:298-304.
6. Baigent C, Blackwell L, Collins R, Emberson J, Godwin J, Peto R, et al. Aspirin in the primary and secondary prevention of vascular disease: collaborative meta-analysis of individual participant data from randomised trials. Lancet. 2009;373:1849-60.
7. Flossmann E, Rothwell PM. Effect of aspirin on long-term risk of colorectal cancer: consistent evidence from randomised and observational studies. Lancet. 2007;369:1603-13.
8. Rothwell PM, Wilson M, Elwin CE, Norrving B, Algra A, Warlow CP, et al. Long-term effect of aspirin on colorectal cancer incidence and mortality: 20-year follow-up of five randomised trials. Lancet. 2010;376:1741-50.
9. Rothwell PM, Fowkes FG, Belch JF, Ogawa H, Warlow CP, Meade TW. Effect of daily aspirin on long-term risk of death due to cancer: analysis of individual patient data from randomised trials. Lancet. 2011;377:31-41.
10. Rothwell PM, Price JF, Fowkes FG, Zanchetti A, Roncaglioni MC, Tognoni G, et al. Short-term effects of daily aspirin on cancer incidence, mortality, and non-vascular death: analysis of the time course of risks and benefits in 51 randomised controlled trials. Lancet. 2012;379:1602-12.
11. Rothwell PM, Wilson M, Price JF, Belch JF, Meade TW, Mehta Z. Effect of daily aspirin on risk of cancer metastasis: a study of incident cancers during randomised controlled trials. Lancet. 2012;379:1591-601.
12. Algra AM, Rothwell PM. Effects of regular aspirin on long-term cancer incidence and metastasis: a systematic comparison of evidence from observational studies versus randomised trials. The Lancet Oncology. 2012;13:518-27.
13. Jacobs EJ, Newton CC, Gapstur SM, Thun MJ. Daily aspirin use and cancer mortality in a large US cohort. Journal of the National Cancer Institute. 2012;104:1208-17.
14. Downer MK, Allard CB, Preston MA, Gaziano JM, Stampfer MJ, Mucci LA, et al. Regular Aspirin Use and the Risk of Lethal Prostate Cancer in the Physicians' Health Study. European urology. 2017.
15. Cook NR, Lee IM, Zhang SM, Moorthy MV, Buring JE. Alternate-day, low-dose aspirin and cancer risk: long-term observational follow-up of a randomized trial. Annals of internal medicine. 2013;159:77-85.

16. ASCEND Collaborative Group. ASCEND: A Study of Cardiovascular Events iN Diabetes: Characteristics of a randomized trial of aspirin and of omega-3 fatty acid supplementation in 15,480 people with diabetes. American Heart Journal. 2017;(submitted).
17. Peto R, Pike MC, Armitage P, Breslow NE, Cox DR, Howard SV, et al. Design and analysis of randomized clinical trials requiring prolonged observation of each patient. I. Introduction and design. British journal of cancer. 1976;34:585-612.
18. Peto R, Pike MC, Armitage P, Breslow NE, Cox DR, Howard SV, et al. Design and analysis of randomized clinical trials requiring prolonged observation of each patient. II. analysis and examples. British journal of cancer. 1977;35:1-39.
19. Yusuf S, Peto R, Lewis J, Collins R, Sleight P. Beta blockade during and after myocardial infarction: an overview of the randomized trials. Prog Cardiovasc Dis. 1985;27:335-71.
20. Cox DR. Regression Models and Life-Tables. Journal of the Royal Statistical Society Series B (Methodological). 1972;34:187-220.
21. Cox DR. Partial likelihood. Biometrika. 1975;62:269-76.
22. Mantel N, Haenszel W. Statistical aspects of the analysis of data from retrospective studies of disease. Journal of the National Cancer Institute. 1959;22:719-48.
23. Collins R, MacMahon S. Reliable assessment of the effects of treatment on mortality and major morbidity, I: clinical trials. Lancet. 2001;357:373-80.
24. Gail M, Simon R. Testing for qualitative interactions between treatment effects and patient subsets. Biometrics. 1985;41:361-72.
25. Mehran R, Rao SV, Bhatt DL, Gibson CM, Caixeta A, Eikelboom J, et al. Standardized bleeding definitions for cardiovascular clinical trials: a consensus report from the Bleeding Academic Research Consortium. Circulation. 2011;123:2736-47.

Appendix 1: Definition of ASCEND event terms

Details of the adjudication procedures used in ASCEND are outlined in the trial Standard Operating Procedures but key definitions are re-iterated here. Any categorisation of baseline information will be based on that in the publication describing the design and baseline characteristics.[16]

Myocardial infarction (MI)

An event is considered to be an MI if there is evidence of cardiac necrosis (consistent elevation in cardiac biomarkers or relevant autopsy findings) and there is other evidence of an acute MI (including symptoms of ischaemia, recent coronary intervention, death, new ECG changes, evidence of a new myocardial defect on cardiac imaging or an acute coronary occlusion at angiography) and no other diagnosis is likely. Silent MI is not included.

Stroke

Stroke is defined as an acute symptomatic episode of focal or global neurological dysfunction caused by brain, spinal or retinal vascular injury as a result of haemorrhage or infarction which lasts >24 hours, leads to death or is associated with evidence of an acute infarct or haemorrhage on brain imaging corresponding with the clinical syndrome. Strokes are further subdivided by aetiology, including confirmed ischaemic, confirmed haemorrhagic or uncertain aetiology.

Adjudication of strokes in ASCEND is based on information provided by the participant's general practitioner, including copies of relevant clinic and discharge letters, therefore information about the level of disability following a stroke may be limited. Stroke events are considered to be disabling if the participants require assistance from another person to perform their activities of daily living and are considered to be non-disabling if no such assistance is required. The assessment of disability is based on the latest information available at the time of adjudication.

Transient ischaemic attack (TIA)

In ASCEND, TIA is defined as a transient episode of neurological dysfunction, lasting less than 24 hours, caused by brain, spinal cord, or retinal ischaemia, without clear evidence of acute infarction, haemorrhage, trauma or another cause.

Coronary and non-coronary revascularisation

Coronary revascularisation includes coronary angioplasty or coronary artery bypass grafting. Non coronary revascularisation includes peripheral angioplasty, atherectomy, thrombectomy, embolectomy, catheter directed thrombolysis, arterial bypass surgery and aneurysm repair (surgical or endovascular). Arterial embolization (e.g. cranial aneurysm coiling or embolization procedures to treat haemorrhage), amputation procedures and procedures on the venous or pulmonary systems are not included.

Cancer

Cancer events are considered incident if that participant is not known to have cancer at that site prior to randomization, or if the cancer was fatal. Site-specific cancer incidence (excluding non-fatal non-melanoma skin cancer) includes: gastrointestinal (i.e. oropharynx, oesophagus, stomach, pancreas, hepatobiliary, ileal and colorectal); respiratory (lung and larynx); genitourinary (GU, i.e. renal, bladder, prostate, gynaecological and other GU); haematological (leukaemia and lymphoma); breast; melanoma skin cancer; other; and unspecified. GI tract cancer is defined as any GI excluding pancreas and hepatobiliary.

Drugs

Cardiovascular and other drugs will be grouped into categories as defined in the baseline paper.[16]

Fatal outcomes

All deaths are adjudicated to ascertain the underlying cause of death by a review of the death certificate and any information about events preceding the death. If, following adjudication, a preceding event is considered to be the underlying cause of the subsequent death, the event is classified as fatal. Deaths will be grouped by underlying cause into: vascular (coronary, stroke, other); non-vascular (cancer, respiratory, other medical, external); and unknown.

Bleeding severity

Bleeding events in ASCEND are recorded by the anatomic site at which the bleeding occurred and extra-ocular bleeding is further subdivided by severity using a

classification based on the Bleeding Academic Research Consortium (BARC) classification (table 1).[25]

Bleeding events are considered to be “serious” if they required hospitalisation or transfusion, or are fatal or disabling (ie ≥4 see below).

Sight-threatening bleeds include clinically significant bleeding in the eye which results in unresolved visual loss and/or requires an urgent intervention such as laser photocoagulation, vitreoretinal surgery or intraocular injections. Visual loss will be considered permanent if it is not known to have resolved using the latest information available at the time of adjudication.

Table 1: ASCEND classification of bleeding by severity

Severity<br>(ASCEND category code)	Criteria for each category (based on a modified version of the BARC bleeding definition)[25]
**1 (M)**<br>**Least serious**	Minimal bleeding that was not actionable and does not cause the participant to seek unscheduled treatment or investigation (participant did not see a doctor for the bleeding event).
**2 (N)**	Bleeding where participant seeks medical advice, but no action was taken, or where it is not possible to determine if any action was taken.
**3 (T)**	Any overt, actionable sign of haemorrhage (e.g. more bleeding than would be expected for a clinical circumstance) where the patient was not admitted to hospital for the bleed and did not meet the criteria for suffixes H, S, L or F, but where the bleeding did meet at least one of the following criteria:<br>- requiring medical or surgical intervention by a healthcare professional,<br>- leading to an increased level of care,<br>- prompting further evaluation or investigation beyond the initial consultation
**4 (H)**	Any overt, actionable sign of haemorrhage (e.g. more bleeding than would be expected for a clinical circumstance) where the patient was admitted to hospital for the bleed, or where the bleeding led to a prolongation of an existing hospital stay, but did not meet the criteria for suffixes S, L or F.
**5 (S)**	- Overt bleeding plus haemoglobin drop[1] of 3-5 g/dL<br>- Any transfusion with overt bleeding
**6 (L)**	- Overt bleeding plus haemoglobin drop[†] >5 g/dL<br>- Bleeding that required surgical intervention for control (excluding minor surgery). [2]<br>- Bleeding that led to the participant being admitted to a high dependency area, or required treatment with vasoactive agents, to manage the bleeding event or sequela.
**7 (F)**<br>**Most serious**	Fatal bleeding[3] (bleeding that definitely or probably led to death).

[1] Provided the haemoglobin drop is related to the bleed.
[2]Within the BARC definition minor surgery includes dental surgery, nasal surgery, skin surgery and haemorrhoid surgery.
[3] Within the BARC definition fatal bleeding is defined as "*bleeding that directly causes death with no other explainable cause.*"

Appendix 2: Derivation of vascular risk score

Poisson regression predicted risk of SVE in ASCEND will be based on age (continuous and 10-year groups), sex, smoking (never/ex; current), baseline SBP (grouped <140; ≥140, <160; ≥160 mmHg; unknown), BMI (grouped <30; ≥30, <35; ≥35 kg per m^2), duration of diabetes (per calendar year), HbA1c measured using the International Federation of Clinical Chemistry [IFCC] method (per mmol/mol at baseline), adjusting for randomized treatment allocation (when unblind).

Participants will be categorised by predicted 5 year risk of SVE without aspirin or omega-3 as low (<5%), moderate (≥5%, <10%) or high (≥10%).

For variables with missing values (except SBP which has a separate category for missing SBP) the median value will be imputed prior to creating the risk score. HbA1c will also include a missing/available indicator variable.

Appendix 3: Censoring rules for analysis of events during the scheduled treatment period

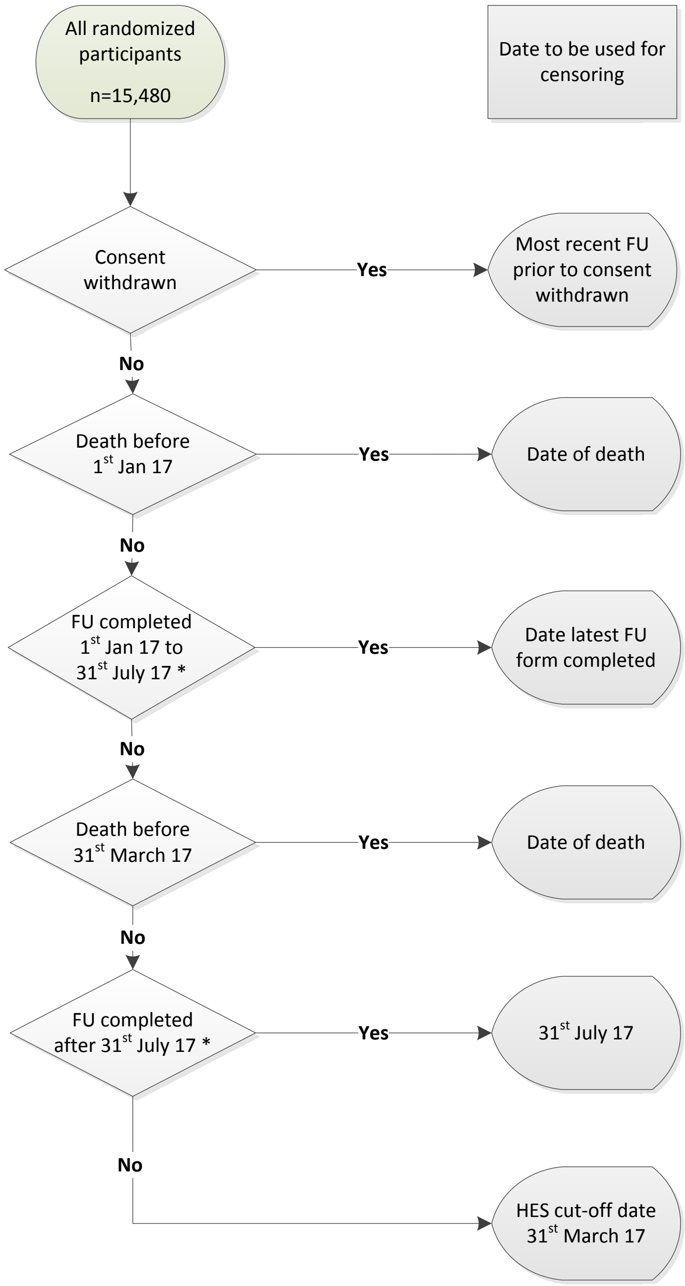

Detailed specification of these censoring rules is contained within the ASCEND analysis specification documents

* by GP or participant

FU; Follow-up: HES; Hospital Episode Statistics
